# Recommendations to Address Barriers to Patient Portal Use Among Persons With Diabetes Seeking Care at Community Health Centers: Interview Study With Patients and Health Care Providers

**DOI:** 10.2196/58526

**Published:** 2024-09-16

**Authors:** Samuel Akyirem, Julie Wagner, Helen N Chen, Joanna Lipson, Maritza Minchala, Karina Cortez, Robin Whittemore

**Affiliations:** 1 Yale School of Nursing Yale University West Haven, CT United States; 2 Behavioral Sciences and Community Health School of Dental Medicine University of Connecticut Farmington, CT United States; 3 Department of Epidemiology, Richard M. Fairbanks School of Public Health Indiana University Indianapolis, IN United States; 4 Center for Biomedical Informatics Regenstrief Institute Indianapolis, IN United States; 5 Yale School of Public Health Yale University New Haven, CT United States

**Keywords:** community health centers, patient portal, type 2 diabetes, self-management, qualitative study, mobile phone

## Abstract

**Background:**

Community health centers (CHCs) are safety-net health care facilities in the United States that provide care for a substantial number of low-income, non-English speaking adults with type 2 diabetes (T2D). Whereas patient portals have been shown to be associated with significant improvements in diabetes self-management and outcomes, they remain underused in CHCs. In addition, little is known about the specific barriers to and facilitators of patient portal use in CHCs and strategies to address the barriers.

**Objective:**

The objectives of this qualitative study were to explore the barriers to and facilitators of the use of patient portals for managing diabetes in 2 CHCs from the perspective of adults with T2D and clinicians (community health workers, nurses, nurse practitioners, and physicians) and to make recommendations on strategies to enhance use.

**Methods:**

A qualitative description design was used. A total of 21 participants (n=13, 62% clinicians and n=8, 38% adults with T2D) were purposively and conveniently selected from 2 CHCs. Adults with T2D were included if they were an established patient of one of the partner CHCs, aged ≥18 years, diagnosed with T2D ≥6 months, and able to read English or Spanish. Clinicians at our partner CHCs who provided care or services for adults with T2D were eligible for this study. Semistructured interviews were conducted in either Spanish or English based on participant preference. Interviews were audio-recorded and transcribed. Spanish interviews were translated into English by a bilingual research assistant. Data were collected between October 5, 2022, and March 16, 2023. Data were analyzed using a rapid content analysis method. Standards of rigor were implemented.

**Results:**

Themes generated from interviews included perceived usefulness and challenges of the patient portal, strategies to improve patient portal use, and challenges in diabetes self-management. Participants were enthusiastic about the potential of the portal to improve access to health information and patient-clinician communication. However, challenges of health and technology literacy, maintaining engagement, and clinician burden were identified. Standardized implementation strategies were recommended to raise awareness of patient portal benefits, provide simplified training and technology support, change clinic workflow to triage messages, customize portal notification messages, minimize clinician burden, and enhance the ease with which blood glucose data can be uploaded into the portal.

**Conclusions:**

Adults with T2D and clinicians at CHCs continue to report pervasive challenges to patient portal use in CHCs. Providing training and technical support on patient portal use for patients with low health literacy at CHCs is a critical next step. Implementing standardized patient portal strategies to address the unique needs of patients receiving care at CHCs also has the potential to improve health equity and health outcomes associated with patient portal use.

## Introduction

### Background

Diabetes remains a significant public health concern in the United States, currently affecting 14.7% of the adult population [1]. The prevalence of diabetes is disproportionately higher among racial and ethnic minority individuals and those with lower socioeconomic status. Diabetes is the 8th leading cause of death in the United States and costs the nation >US $410 billion in both direct and indirect costs [1]. The disease can lead to microvascular and macrovascular complications that may increase the risk of morbidity and mortality as well as reduce the quality of life of persons living with the disease [2]. The American Diabetes Association recommends that persons with diabetes be supported to engage in self-management behaviors, including healthy eating, taking medication, glucose self-monitoring, healthy coping, and physical activity. These behaviors have been shown to improve glycemic control, psychosocial outcomes, and delaying or preventing complications [3].

Patient portals are increasingly recognized as important tools to support diabetes self-management [4]. Patient portals are digital platforms that allow patients to access their electronic health records (EHRs) to view their health information and securely communicate with their health care team. Patient portals vary, but common features allow patients to request appointments, view laboratory findings, request medication refills, view medication lists, and exchange nonurgent messages with clinicians [5].

Patient portal use is associated with significant improvements in diabetes self-management behaviors and outcomes. Studies have shown that users of patient portals, compared to nonusers, are more likely to meet glycemic targets and have better lipid profiles [6-8]. In a retrospective cohort study among 95,043 persons with diabetes, individuals who used the patient portal every month of the year had 0.41% lower hemoglobin A_1c_, 6.25 mg/dL lower low-density lipoprotein, and 1.01 mmHg lower systolic blood pressure compared with those who used the patient portal for only 1 month in a year [8]. Previous qualitative studies show that patient portals provide patients with easy access to timely information [5,9-11], foster family involvement in diabetes care [12], improve the quality of patient-clinician communication [13-15], and help to facilitate lifestyle changes [10,16]. For instance, in 1 study, persons with diabetes reported that the ability to access their laboratory results and notes from previous visits enabled them to track changes in their overall health, and thus, empowered them to take charge of their disease management [17].

Despite the known benefits of patient portals, disparities exist in their access and use. Patient portal use is significantly lower among Black individuals, Hispanic individuals, and Asian individuals living with diabetes compared to their White counterparts [18-22]. In a cross-sectional study of >38,000 persons living with diabetes, individuals with low income and those living in rural areas were less likely to use patient portals [23]. Low literacy and low educational attainment have been linked to a lower likelihood of adopting patient portals among persons with diabetes [19,24]. There is evidence of low patient portal use among patients with Spanish as their preferred language. In a retrospective study, Spanish speakers in safety-net clinics were less likely to receive a patient portal access code, activate an account, and use the patient portal more than once in 2 years compared to English speakers [25]. This disparity is likely at least partly attributable to the limited number of bilingual (English and Spanish) patient portals in the United States which means that individuals with limited English proficiency are practically excluded [12,26]. Despite their advantages, patient portals remain underused in the populations that need them most.

Several studies have examined the barriers to and facilitators of patient portal use among persons living with diabetes [5,10,13,16,27]. Barriers include poor internet connection [10], limited computer knowledge [5,10,27], being unaware of patient portal features [13,16], privacy concerns [10,27], and not feeling the need to use the patient portal when health outcomes are perceived as stable [10]. In other studies, participants report that the need to maintain face-to-face interaction with their clinicians discourages the adoption of patient portals [27]. Persons with diabetes are more likely to use the patient portal if they are dissatisfied with the diabetes-related information they receive from their clinician, thus, resorting to the patient portal as an additional source of information [11]. Patient portal use also increases when caregivers or family members are involved in helping patients with the portal and detailed instructions on use are provided to patients and their families [12,27].

Whereas perspectives from some patient populations have been studied, there is limited qualitative evidence from low-income and non-English speaking individuals with diabetes in the United States. Non-English speakers and low-income individuals have limited access to diabetes self-management support due to language barriers and are at a higher risk of diabetes complications. In addition, fewer studies incorporate the views of clinicians to obtain a complete picture of the challenges of patient portal use and recommendations to address them. In the United States, community health centers (CHCs), formerly called federally qualified health centers [28], provide care for a substantial number of low-income and non-English speaking individuals. CHCs are safety-net health care centers that offer outpatient services at a lower cost to more than 30 million individuals in the United States. The aims of CHCs are to provide high quality, comprehensive primary care to medically underserved populations, regardless of insurance status or ability to pay for care. Most CHC patients (90%) live in poverty or near poverty as defined by the federal poverty level, are disproportionately from racial and ethnic minority groups (total 63%) and have high rates of chronic conditions compared to the general population [29]. The uptake of patient portals in CHCs is limited especially among individuals with limited English proficiency [25], and few studies have examined specific barriers and facilitators among patients seeking care at these facilities [27]. Given the benefits of patient portals in improving glycemic control, understanding the specific facilitators of and barriers to portal use in this population considered vulnerable is crucial. Such data can aid in the design of interventions to improve access to and use of portals, with the end goal of improving health equity and health outcomes.

### Objectives

The purpose of this qualitative study was to explore the barriers to and facilitators of the use of patient portals for managing diabetes in 2 CHCs from the perspective of adults with type 2 diabetes (T2D) and clinicians. Strategies to enhance patient portal use in CHCs were also explored. The data reported in this study were collected as part of a larger study to design and pilot a multilevel, bilingual intervention to improve patient portal use among low-income individuals with diabetes receiving care at CHCs.

## Methods

### Study Design and Setting

A qualitative description design, a well-established qualitative method, was used to describe the perceptions of adults with T2D and clinicians regarding diabetes self-management and patient portal use [30].

The study was conducted in 2 urban CHCs in Connecticut. Connecticut is a small and densely populated state with prominent health disparities. Black Connecticut residents are nearly 4 times as likely as White residents to have a diabetes-related lower extremity amputation, and the rate is nearly 3 times higher among Hispanic individuals compared to non-Hispanic White individuals [31]. One CHC has 2 clinical sites that provide care to approximately 32,000 residents (including 12,600, adults), 62% Hispanic, 45% Black, 10% report as more than 1 race, and 15% White, with 1763 adults diagnosed with T2D [32]. Similarly, the other CHC has 2 clinical sites that provide care to approximately 20,000 residents, 66% Hispanic, 16% Black, 11% White, 3% Asian, with 2095 diagnosed with T2D [32].

At 1 clinic, most health care providers use the patient portal in some capacity; in the other clinic, few clinicians use the patient portal as they recently changed to a new EHR and patient portal system. Patient portal features at our partner CHCs include the ability for patients to make appointments, refill medications, view test results, and in 1 clinic, securely message clinicians. In both clinics, primary care providers manage T2D, and access to an endocrinologist is available. T2D education is provided by primary care providers and nurses as needed.

### Recruitment

We used convenience and purposive sampling to recruit study participants. Participant recruitment and data collection took place toward the end of the COVID-19 public health emergency between October 5, 2022, and March 16, 2023. We recruited participants through Spanish and English flyers in participating clinics, in-person recruitment by research assistants (RAs), and recommendations from clinic leadership and participating stakeholders. Members of the research team assessed eligibility and obtained written informed consent from patients in their preferred language, and clinicians provided verbal consent.

Inclusion criteria for patients were as follows: established patient of 1 of the partner CHCs, aged ≥18 years, diagnosed with T2D ≥6 months, and able to read English or Spanish. For patients, we sought variability in age, sex, race, ethnicity, language (English or Spanish), type of insurance, duration of T2D, and current portal use. Inclusion criteria for clinicians were as follows: clinician or community health worker (CHW) at 1 of our partner CHCs and provided care or services for adults with T2D. For clinicians, we sought variability in age, sex, and type of clinician.

### Data Collection

Semistructured interviews were conducted by trained bilingual RAs. Interview guides were collaboratively developed by the research team to assess the individual, social, health care system, and health inequity challenges related to patient portal use for T2D management (Textbox 1). Interviews were conducted via a video call platform (Zoom; Zoom Video Communications, Inc). All interviews were audio-recorded and transcribed verbatim via the video call platform. Spanish transcripts were translated into English, and all transcripts were deidentified and checked or corrected for accuracy by bilingual RAs. Participants also completed a brief demographic questionnaire. All participants were assigned a code number, and names were not transcribed in any interviews to assure confidentiality. Participants continued to be recruited until information saturation was achieved.

**Interview guide.**

**Interview questions**

- What are the most difficult challenges in caring for adults with diabetes in this clinic? (clinicians) What challenges do you have in taking care of your diabetes? (adults with type 2 diabetes)
- Describe your experience and thoughts about using the patient portal.
- What are the barriers and facilitators to your use of the portal? Patients’ use of the portal? (clinicians)
- Elicit feedback on the proposed components of intervention.
- How could a nurse help you and your patients (clinicians), using the portal?
- Anything else you would like to share?

### Data Analysis

Data were analyzed using a rapid content analysis method [33,34]. A codebook was developed a priori based on the interview guide categories. Research team members, in teams of 2, independently coded and extracted data into the coding categories. Coding discrepancies were resolved within teams or by review by the primary investigator (RW). A second review of the data extraction was conducted by 2 research team members (RW and HNC), who met frequently during the coding process to develop coding categories, memos, and the overarching conceptualization of themes, using Microsoft Word and Excel. Most categories were endorsed by clinicians and adults with T2D; however, there were several subcategories unique to each group. To ensure methodological rigor, we re-examined transcripts to confirm that the coding process was reflective of participants’ perspective. We maintained an audit trail of all coding decisions and memos and used a consensus, collaborative approach to finalize the data analysis process and final conceptualization. The results of this study informed the development of an intervention protocol for a multilevel intervention to promote patient portal use in adults with T2D who access care at the partner CHCs.

### Ethical Considerations

Institutional Review Board approval was obtained from Yale University (IRB# 2000031753; approved on December 21, 2022). The Consolidated Criteria for Reporting Qualitative Research was used to guide the reporting of this study [35]. Participants received a gift card for US $20.00 for their time.

## Results

### Sample Description

The sample included 13 (62%) clinicians and 8 (38%) adults with T2D (N=21). All clinicians contacted completed interviews. Of the 14 adults with T2D approached for this study, 4 (29%) declined participation (too busy or not interested), and 2 (14%) were unable to be reached after the initial contact (8/14, 57% participation rate). Participating adults with T2D were aged 61.13 (SD 7.06; range 53 to 72) years and consisted of 62% (5/8) women and 38% (3/8) men. Individuals from minority racial and ethnic groups comprised 88% (7/8) of the sample (Hispanic: n=5, 62%; Black: n=1, 12%; and Asian: n=1, 12%), and 12% (1/8) non-Hispanic White. In addition, 50% (4/8) of participants were interviewed in English and 50% (4/8) in Spanish. Only 25% (2/8) of participants endorsed having medical insurance; 75% (6/8) reported no insurance. Time since T2D diagnosis varied from 6 to 35 years. Self-reported hemoglobin A_1c_ values ranged from 7% to 9% (SD 0.84%). The length of engagement with the CHC spanned from 1 to 17 years. Only 3 (38%) of the 8 participants had ever used a patient portal. For clinicians, age ranged from 25 to 72 years, with a mean of 47.46 (SD 15.93) years, and the majority were female (11/13, 85%). Clinicians were 62% (8/13) non-Hispanic White, with 38% (5/13) Hispanic. The type of clinicians included nurses (5/13, 38%), physicians (n=2, 50%), nurse practitioners (n=2, 50%), and CHWs (2/13, 15%). Interviews ranged from 12 to 32 minutes for adults with T2D and 20 to 54 minutes for clinicians.

### Qualitative Themes

#### Overview

Factors influencing the integration of patient portal use in CHCs included challenges in diabetes self-management, perceived usefulness of the patient portal, and challenges in using the patient portal. Strategies to enhance patient portal use were also identified by clinicians and adults with T2D. Each of these themes is described in more detail in the subsequent sections.

#### Challenges in Diabetes Self-Management

Clinicians and adults with T2D described numerous general challenges to diabetes self-management. These included addressing social determinants of health (SDOH), complex self-management of T2D, and management of comorbidities, some of which could be improved by greater use of the patient portal. Clinicians and adults with T2D both described SDOH factors of social status, low socioeconomic position, lack of material resources (eg, access to medications and food insecurity), lack of insurance, language, culture, and health literacy as barriers to performing self-management behaviors, including engagement in technology such as a patient portal. Adults with T2D and health care providers also reported that the complex and long-term trajectory of T2D was challenging. Adults with T2D reported difficulty with fluctuating blood glucose (BG) levels and hyperglycemia despite self-management efforts. Several adults with T2D reported that additional health problems or pain made diabetes self-management difficult. In addition, several adults with T2D reported feelings of frustration, being overwhelmed, distress, and depression as a result of diabetes self-management.

#### Patient Portal Use and Perceived Usefulness (Facilitators)

While both of our CHCs were using patient portals, 1 clinic had recently adopted a new EHR, and the patient portal interface had only been activated in a limited capacity (eg, for making appointments) or with select clinicians. In our sample of clinicians, all (4/4, 100%) physicians currently used the patient portal, only 14% (1/7) of nurses, and no CHW used the portal. Of adults with T2D, 25% (2/8) used the patient portal, and 25% (2/8) had tried to use the patient portal without success. Across our study participants, 33% (7/21) were currently using the patient portal. Despite low use of the patient portal, most adults with T2D and clinicians reported perceived usefulness of the patient portal. Adults with T2D and clinicians reported that access to smartphones and internet capabilities was available to most people, if not individually, then through a shared device or with support from family members. Potential benefits of the patient portal endorsed by adults with T2D and clinicians included the ability to schedule appointments more efficiently, obtain refill prescriptions for medications, and access health information quickly, such as laboratory or test results and health care visit summaries. One adult with T2D stated the following:

I can look like the very next day and see my blood results.

Adults with T2D and clinicians also identified the ability to communicate between health care appointments, ask or respond to a nonurgent question, and have communication in between appointments as clear benefits to the use of the patient portal. As 1 adult with T2D stated the following:

The portal would be good, because then you don’t have to go stepping out of your house in order to ask a question...you have somebody there to advocate for you.

Specific to T2D self-management, several adults with T2D and clinicians perceived that the patient portal could be useful for maintaining motivation and having support for the challenges of diabetes self-management. One adult with T2D stated, “It’s really good motivation to be aware of what’s going on,” referring to the patient portal as a way to increase awareness about diabetes care. One clinician stated that through the patient portal, we may be able to address “things that unfortunately the provider and their fifteen minutes may not emphasize, things like behavioral health, immunizations, dental, things that unfortunately often get by the wayside.” For example, this clinician indicated that a yearly information sheet could be sent to all patients with diabetes, reminding them to schedule health maintenance appointments. Other educational resources about coping with diabetes and behavioral health resources could also be sent via the portal.

#### Patient Portal Challenges

##### Overview

Whereas adults with T2D and clinicians described the potential usefulness of the patient portal as an adjunct to T2D care in a CHC, numerous challenges were identified. This included a lack of engagement in the use of the portal by patients and clinicians, low socioeconomic status, language, literacy (including technical literacy), and patient portal factors related to design, usability, and implementation specific to CHCs. Neither clinic had a current practice for training patients or clinicians in the use of the portal, rather it was clinician-directed.

##### Lack of Engagement in Portal Use

Adults with T2D and clinicians shared that patient portal use was underused in their health care setting. Clinicians may not recommend use or have experience in patient portal use, and adults with T2D may not be enrolled or, if enrolled, do not use the patient portal consistently. Factors contributing to lack of engagement include perceived clinician burden, competing demands for adults with T2D, or lack of technology support for adults with T2D in patient portal use. One clinician stated the following:

Checking a portal twenty-four seven is not something that any of us want to do.

With regard to engagement, another clinician stated the following:

I think the biggest challenge or barrier would be getting the patient engaged to actually download the application and manipulate it and understand how to use it as a tool.

Adults with T2D reported a lack of time, competing demands, and the need for technology support as barriers to engagement with the patient portal. In reference to navigating the patient portal, 1 adult with T2D stated the following:

You have to get over the learning curve.

Other adults with T2D reported preferring in-person communication with their health care team while clinicians reported that they have other established patterns of communication between health care appointments with phone calls and SMS text messaging.

##### Socioeconomic Status, Language, and Literacy

Participants reported that although many adults with T2D had access to smartphones and community-based internet, access to the internet at home or tablets or computers with larger screen sizes may not be available at home, limiting timely access or easy reading of small font sizes. One adult with T2D stated the following:

I’m drowning with that price for internet and cable.

Clinicians reported that language or literacy barriers may be exacerbated in patients’ understanding and ability to respond to written messages in the patient portal (whether in English or Spanish). Most adults with T2D and clinicians identified challenges associated with technical literacy in using the patient portal which requires downloading the application, creating an account using an email address, creating, and remembering how to enter a password, navigating the patient portal interface, and entering data or written information. As 1 adult with T2D shared the following:

I went there to the clinic...they downloaded it for me, they entered an email...I had to enter it for them to download it, but it does not work, it does not show up...I have tried, but no. I cannot use it.

Another adult with T2D stated the following:

I’ve been left behind with this technology. I haven’t learned English well. I’ve lost all my time working. I’ve been here for more than 20 years. I’ve only been dedicated to 2 jobs. I tell my daughters to do it for me.

##### Patient Portal Design, Usability, and Implementation Factors

Clinicians shared numerous portal design, usability, and implementation factors that were challenges to the widespread adoption of patient portal use in CHCs (Textbox 2). Patient portal design issues included features and options available. Clinicians reported that the portal’s overall design with the requirement for manual data entry, typing of text or BG values, could be challenging for adults who may have low educational attainment. Spelling and grammar to articulate health concerns may be difficult for some patients. Navigating the portal interface to determine how to enter each BG data point may not be user-friendly, all the more for adults with limited health and numerical literacy. Clinicians also reported that patients had difficulty accessing or using patient portal features that allowed them to message their health care team:

When I sign off on labs or imaging there is a spot for me to put notes that the patient can view; but there’s no opportunity for the patient to ask a question back.

**Summary of the challenges of patient portal design, usability, and implementation.**

**Design**

- Document upload and data not interoperable for electronic health record
- Unable to upload images
- Requires manual entry
- Limited messaging options (eg, unable to post a question on a test result)
- Cost for certain features to community health centers (eg, alerts)

**Usability**

- Clinic workflow, linkage between patient and correct clinician for a given message
- Patient users need an alert for when messages or health information sent
- Data entry can be challenging for patient users
- No synthesis of blood glucose data useful for clinicians (eg, data trends or patterns for drug treatment adjustment)

**Implementation**

- Inadequate staff to triage messages
- Inappropriate message content that cannot be addressed via the portal
- Disparity in the expectation of response time by both users (patients and clinicians)
- Lack of clear procedures for unanswered messages by both users
- Clinician concern about medical decision-making
- Clinician may need objective data or in-person appointment
- Clinician time or burden, currently not reimbursable

Patient portal usability issues included navigation, interface, and tasks required for both users (patients and clinicians). One clinician stated the following:

You can only send (a message) to certain providers, and I don’t know how do you set it up to send it to a different provider?

Implementation issues included inadequate staff for triaging messages, inappropriate use of messaging, long response time, decision-making with limited information, clinician time or burden, and the issue of reimbursement for clinician time. For example, 1 clinician stated the following:

The patient will say this, and then the provider will send them a message back. But whatever was true three days ago is not true anymore.

Another clinician stated that she did not make medication changes through the patient portal as she was concerned patients will not see the message.

#### Strategies to Enhance Portal Use

A total of 3 broad themes emerged regarding strategies to enhance patient portal use in CHCs: (1) standardized clinic- and patient-level implementation strategies to integrate use into clinic workflow, (2) technical modifications tailored for clinicians and adults with T2D, and (3) patient-centered chronic illness management to address SDOH, mental health, and psychosocial support needs.

##### Standardized Implementation Strategies

Standardized clinic-level strategies included an implementation guide on the specifics of clinic workflow procedures, individual roles, and responsibilities. Clinicians questioned who would lead clinic-level implementation. For example, 1 clinician stated the following:

If we’re going to do this, implement this truly,...we do need to rethink our systems and evolve toward systems where we have other people who are...who are triaging those things? I think it’s the big thing.

Personnel challenges at the clinic were identified, such as high turnover for medical assistants or CHWs, registered nurses, and physicians who gain experience and then move on to other settings. One clinician stated the following:

It’s impossible to hire nurses.

Despite these challenges, nurses were recognized as a key clinician to triage health-related concerns on the patient portal. For example, 1 suggestion was having a nurse as the initial contact with a decision algorithm detailing: (1) what message to respond to versus route to an appropriate recipient, (2) response time (eg, general questions vs real-time high or low BG), (3) conditions for nurse video visit or other health care provider appointment, and (4) preset templates of messages to be consistent and efficient. One clinician stated the following about the number of messages:

How do you handle 3-400 all the time—high volume needs.

Clinicians felt that an algorithm should give guidance from the beginning of a message to subsequent ones and include how to handle critical laboratories or clinical issues, such as hypoglycemia.

Standardized patient-level strategies included an implementation guide with steps to support patient engagement. The key emphasis was to educate patients about the importance of messaging via the patient portal to build long-term patient-clinician relationships and trust, with regular check-ins. Clinicians recommended that time at the end of clinic visits could be used for specified clinic personnel to teach and show patients how to download and use the patient portal application, allowing enough time for patients to learn the technology. One clinician stated:

I think that it’s just having a lot of training in that sense and then also when it comes to medical terms on the patient portal, I think that that could get a little tricky for them. It’s going to take a lot of kind of reinforcement and just I would think definitely a lot of training for some...individuals.

Clinicians expressed the need for a training plan for patients and the health care team. There were suggestions for teaching patients about the patient portal, features or navigation, how to use it (login, see laboratories, send, and receive messages), and logistics (eg, who does what on the health care team, how frequently messages are checked or responded to). Training recommendations for adults with T2D included having bilingual personnel and a combination of in-person training with telephone or internet-based training options available to accommodate patient scheduling needs. One clinician stated the following:

Here you go...don’t go through the whole thing...I think that it would be beneficial to actually have a little bit of time and send a message and show them how to send a message back.

Reinforcement of training and having technical support staff was also deemed important. One adult with T2D stated that sessions needed to be frequent:

Because you can’t (have) too much time go in between, because they’re going to forget. You have to make it so that it’s a cumulative thing of knowledge...so they don’t lose anything in the meantime.

Another adult with T2D stated that “it will maybe take 3 or 4 times” (to learn to use the patient portal).

##### Technical Modifications

Participants shared their recommendations on key technical designs tailored for clinicians and patients on data sharing, electronic patient-friendly education material, clinic visit items, alerts or reminders set up, social support resources, and behavioral health referrals. Several clinicians expressed that they would like to have easier interfaces for patients to upload BG results and easier access to simple patient education materials electronically on topics such as healthy eating, exercise, glucose targets, laboratory results, hypoglycemia, medication side effects, medication management, insulin use, and recipes. Suggestions were expressed to provide access to prior clinic visit summaries and care plans in the patient portal. Another suggestion among clinicians was having individualized alerts and reminders on sharing BG data, continuous glucose monitoring (CGM) technique, preventive care visits (eg, specialists), responses or nonresponses to patient portal messages, and when a follow-up for laboratory or clinic was past due. One clinician felt that the patient portal could be an efficient and effective tool to follow up on recommendations made in a clinical visit, both for clinicians to remind patients and for patients to report back to clinicians. This clinician stated the following:

Patients forget to send in 2 weeks of CGM and glucometer BG readings requested by provider—Need reminders to do action, wished MyChart could send alert to patients to share data.

A few clinicians suggested a section for social support resources on the patient portal.

##### Patient-Centered Chronic Illness Management

Most clinicians and adults with T2D expressed a need for patient-centered chronic illness management to address SDOH, mental health, and psychosocial support needs. Adults with T2D expressed that the patient portal held great potential to support regular contact and share concerns, challenges, or successes affecting their health. Adults with T2D also stated that they needed more health education on T2D management that could be done via the patient portal, particularly around glucose variability. One adult with T2D stated the following:

It is so tricky, one time it’s up, one time it’s down...sometimes I’m overwhelmed, and I don’t know what to do.

Spanish-speaking adults with T2D also mentioned that cultural factors, such as foods and celebrations, affected diabetes self-management, and language barriers impacted their understanding of diabetes.

Clinicians felt that the patient portal could be used to provide advice, personal coaching, and encouragement around diabetes self-management or follow up to changes in treatment. Problem-solving for changing health status and mental health were also expressed as challenges that could be addressed on the patient portal. For example, 1 clinician spoke about how the portal could be used to stay connected with patients and identify the need for changes in treatment:

Almost everybody (with a chronic illness)...has issues that they need to work through...through a lifetime, because it’s dynamic right. Nothing stays the same, your life doesn’t stay the same, so nothing is the same.

This clinician also felt that the patient portal could be helpful to support coping with the challenges of self-management:

Whenever, whatever it is, you know, they got a lot of other things going on their lives like everybody else. But...finding a way to cope...and being able to...navigate their chronic illness.

This clinician went on to state that the portal could also be used to refer and follow-up on referrals to behavioral health:

I think...I would include behavioral health...a strong behavioral health program where even the nurse could make a referral.

Another clinician spoke about the benefit of ongoing connection through the patient portal to “follow up for any previous content/discussion—did it work or not? Were you able to do that? Follow up to affirm or deny, the success of the process. In a supportive way. This is life. This is difficult.”

## Discussion

### Principal Findings

In this study, we identify that both patients and clinicians report that patient portals have the potential to facilitate patient-clinician communication, serving as a mechanism through which the health care team can provide diabetes self-management support. However, numerous barriers to patient portal use were also reported, including language, literacy, and low socioeconomic status of patients, as well as design-related challenges to patient portal use and suboptimal implementation of portals in CHCs. Patients and clinicians shared recommendations to overcome these barriers and facilitate portal use, including providing portal education, hands-on training, ongoing technical support, and having standardized implementation strategies for patients and clinicians.

Although several studies have been published on barriers to patient portal use, the study that is most germane to our findings was published in 2015 [27]. Some improvements have been made in the intervening 9 years since this 2015 study was published. These improvements include a substantial increase in access to both smartphones and the internet in the United States from 59% in 2015 to 85% in 2021 [36]. In addition, patients and clinicians still see the potential for patient portals to improve diabetes self-management and outcomes. Yet, it is important to highlight that many challenges to patient portal adoption in CHCs stubbornly persist. Challenges include the lack of adequate training and technical support for patient portal use, particularly for those with low health or technical literacy and the lack of patient-level and clinic-level standardized and thoughtful implementation strategies to address the unique needs of patients receiving care at CHCs.

Our study extends prior work in several important ways. First, we interviewed both patients and a variety of clinicians (eg, nurses). Whereas patients and clinicians did not contradict each other per se, each group of stakeholders did provide a unique perspective, both of which are important for our understanding of patient portal uptake in CHCs. Second, we included Spanish speakers who may face unique challenges with using patient portals designed in English. Third, we examined patient portals in the context of CHCs where the prevalence of diabetes is higher than the general population (21% vs 11%) [29]. Finally, we elicited strategies to increase patient portal use in a CHC setting which may be different from strategies in other health care settings. The findings from this study have the potential to enhance the development of patient and clinic-level portal implementation strategies and to strengthen other initiatives currently underway in CHCs regarding SDOH screening, referral, and support [37].

### Perceived Benefits of Portals

In this study, both clinicians and adults with T2D recognized the usefulness of patient portals in creating an opportunity for adults with T2D to share their successes and nonurgent concerns and for clinicians to provide encouragement or advice on how to address concerns between appointments. In a similar study among adults with diabetes in a safety-net hospital in San Francisco, it was reported that the secure messaging feature of patient portals allowed adults easy access to their health care team and reduced their need for frequent clinic visits [10]. Access to one’s health care clinician outside clinic appointments is associated with better patient-clinician communication and a greater likelihood of patients following diabetes self-management recommendations [38]. Consistent with the findings from this study, the ability to contact clinicians outside of clinic appointments is particularly useful given the limited time clinicians have to spend with patients during outpatient visits [39]. Thus, our study affirms that the findings from previous studies regarding the perceived benefits of portals are also recognized in the CHC setting [10,13,14,16,40].

Clinicians and adults with T2D also reported that the ability to view laboratory results and medications, and access health information on patient portals can contribute positively to their self-management. When adults with T2D have access to their personal health information, they are more likely to achieve their glycemic targets, experience greater self-efficacy, and report feeling more empowered to manage their diabetes [41]. In addition, intervention studies have shown that providing patients access to their medical information can result in a greater likelihood of taking medication as recommended [42]. By having access to personal medical information, adults with T2D can review clinicians’ recommendations for diabetes self-management as frequently as needed. The importance of having access to health information is underscored by Healthy People 2030, which identifies the provision of online access to EHR and other health information as a critical goal aimed at bolstering health literacy and improving health outcomes [43]. Enhancing patients’ access to EHR via patient portals may help address some dimensions of health literacy, which is generally low among individuals who access care at CHCs [44,45]. For example, patient portals may promote patients’ information-seeking habits, particularly if information is simplified, in easy-to-understand language. The use of patient portals also facilitates the use of web-based resources, including easy-to-understand videos (eg, resources from CDC [46]). Thus, taken together, patient portals have the potential to facilitate self-management among adults with T2D who access care at CHCs.

### Patient-Level Barriers to Portal Use and Recommendations to Address Them in CHCs

Both clinicians and patients in this study reported patient-level barriers, such as limited English language proficiency and low technology literacy that interfere with participants’ ability to use portals. These SDOH-related barriers have been well-documented in prior research [4,16,27]. For instance, a recent systematic review of patient portal use among adults with chronic diseases found consistent evidence that individuals with low health literacy and limited access to a computer were less likely to use patient portals [4].

Our study participants proposed several strategies to address these patient-level challenges. Recommendations included addressing SDOH needs prior to initiating patient portal education and training. Providing flexible patient portal education and training with ongoing technical support was deemed essential. These recommendations are consistent with prior studies among adults in non-CHC settings [47]. In 1 study, 48% of patients with chronic conditions who received training on using the patient portal registered for the portal, compared to only 11% of those who did not receive any training [48]. Technical training on patient portals may include setting clearer guidelines on how adults with T2D can use the messaging features [49]. Studies among populations considered vulnerable (such as racial-ethnic minorities and individuals with low literacy) show that providing technical training in patient portal use leads to significant increase in the adoption and use of patient portals [50]. Ongoing technical support may also be required to sustain the continuous use of patient portals among adults with T2D [51]. It should be noted that the population of adults with T2D who access care at CHCs are likely to be socioeconomically disadvantaged individuals who may have barriers to additional clinic visits for training or education [29]. It is, therefore, important for training to be designed and delivered in a flexible manner to accommodate common life circumstances, such as shift work and child or older adult care responsibilities. As suggested by our study participants and in alignment with current literature, strategies may include an initial in-person guide on how to use the patient portal [52], followed by synchronous or asynchronous web-based video tutorials [53,54] phone call and message reminders [55,56], or clinic-specific infographics on patient portal use, problem-solving, and accessing technical support.

In addition, assessing health literacy is important to identify adults with T2D with limited reading or writing literacy who may not benefit from use of the patient portals and to identify adults with T2D who may need additional support or more extensive patient portal training [57]. The health literacy screener question from the Confidence Completing Medical Form tool could be used as a simple and easy-to-use survey to assess health literacy [58] as it can be administered rapidly and has been shown to be sensitive in identifying adults with limited to extremely low health literacy in CHCs [58]. Some practical, evidence-based approaches to accommodate low-literacy adults when delivering patient portal training at CHCs may include avoiding unclear statements or medical jargon, keeping training sessions brief, using bilingual instructors, providing opportunities for patients to practice using the patient portal between sessions, and using a friendly tone [59]. The teach-back method may also be a helpful technique. It involves providing adults with T2D with the most relevant information regarding the topic, with the aid of visual tools, and confirming patients’ understanding by having them describe the information they have been taught, using their own words [60]. In the case of patient portal use, it may also involve patients showing that they can execute a specific task. The teach-back method has been effective in improving knowledge recall and retention among adults with low health literacy [61]. For patients with low technical literacy, a strategy to improve portal use, in addition to ongoing technical support, is to provide proxy access of portals to patients’ caregivers or family members, as this can potentially lower patients’ concerns about self-efficacy in using the portal [57]. However, any concerns about data privacy and control should be carefully navigated when implementing proxy access [62].

### Clinic-Level Barriers to Portal Use and Recommendations to Address Them in CHCs

Whereas both clinicians and adults with T2D expressed shared barriers to portal use, such as limited technology literacy and the need for technical support or training, clinicians also reported their own unique barriers. Notably, clinicians expressed concerns about the lack of reimbursement for the time they spend communicating with patients via the patient portal. Indeed, a cross-sectional study among clinicians (physicians, physician assistants, and nurse practitioners, N=59) in a Spanish-speaking safety-net hospital reported that 64% of clinicians were concerned about the lack of reimbursement for using patient portals [63]. However, billing for patient portal messaging is a complex issue [64]. Whereas such billing might help compensate clinicians for their time, it may also present financial barriers to patients with low income who access care at CHCs [65]. Indeed, there is evidence that when patients know that their interactions on patient portals are billed, they are less likely to message their health care team [66]. Practical strategies to equitably introduce patient portal reimbursement may include waiving copays for sending messages via portals and closely monitoring reimbursement policies to determine their effects on patient experience and health outcomes and making necessary changes as required [64].

In addition, clinicians reported a lack of clear instructions to guide their interaction with patients. Studies have shown that the lack of clear “rules of engagement” when interacting with patients via the patient portal makes it challenging for clinicians to adopt patient portals as a desired platform for communicating with patients [67]. Clinicians in our study suggested that the implementation of patient portals in CHCs should be standardized by providing a clear decision-making algorithm to guide the triaging of messages and clinician responses. The use of an algorithm helps to ensure that adults with T2D have consistent experience with the patient portal regardless of which clinician they are interacting with [57]. In a survey of 1417 frontline workers in 54 CHCs across the United States, about 40% of study participants suggested that having a standardized clinical workflow and operational guidelines can positively contribute to improved care at CHCs [68]. Clinicians may be less likely to find the use of patient portals burdensome if there are clear and easily referenced guidelines for their use.

Staffing challenges and turnover contributing to increased workload are well-documented at CHCs [69] and were reported as a barrier to patient portal use by clinicians in this study. Improving retention of staff can improve care delivery at CHCs [68], yet it remains a challenge. As suggested by our participants, another strategy to reduce clinician workload and burnout in relation to portal use in CHCs is to implement a triaging system to manage messages sent by patients via patient portals. Studies have shown that clinicians can receive excessive messages (eg, form requests and referral responses) via portals that can lead to burnout and contribute to job dissatisfaction [70]. Practical steps to address this information and work overload at CHCs may include having dedicated nursing staff who review messages sent via patient portals, address health questions or concerns within their scope of practice, and forward only messages that require the expertise of primary care providers [71]. In more recent research, artificial intelligence (AI) technology, such as natural language processing, has been used to identify, prioritize, and route urgent patient messages to clinicians. Although natural language processing triaging has been shown to significantly reduce the burden associated with portal use among clinicians [72], the technology is still emerging and requires further testing especially given the concerns of hallucination (a phenomenon where AI generates a convincing but completely fabricated output) in AI technologies and the danger it may pose to patients [73].

Currently, there are a few national interventions, such as the Medicaid EHR Incentive Program, which aims to encourage health facilities to adopt EHR and patient portals [74]. Although this program has contributed to the widespread availability of patient portals across health facilities in the United States [75], it is clear that health facilities that serve underserved populations still face challenges in using this technology in clinical care. Thus, future national programs should prioritize specific incentives and resources that promote equitable access to patient portals for minority populations.

### Portal Design Barriers and Recommendations to Address Them in CHCs

In our study, adults with T2D and clinicians reported challenges navigating specific features of the patient portal. These usability challenges, including difficulties in uploading BG results and the lack of notifications when new health information is available, have been reported in previous studies as important design-related barriers to patient portal use [5,13,19]. For instance, in 1 study, adults with T2D reported difficulty in intuitively understanding the patient portal layout and in using its navigation menu to explore features, making it challenging to continue using the patient portal [76]. In addition, the lack of notification features on patient portals can lead to lapses in communication between adults with T2D and their health care team. Customizing patient portal notifications to meet the needs of clinicians and patients is essential. For example, patients may prefer getting SMS text messages for new messages on the patient portal as they do not login regularly or use email, while clinicians may prefer a notification of messages on their clinical dashboard.

Additional recommendations to address patient portal design and usability barriers included the improvement of features for the target population. These improvements include facilitating the seamless upload of BG results, developing alert systems, expanding access to personal medical information such as visit summaries, and increasing access to resources on improving psychosocial well-being. A previous study reported that 86% of adults with diabetes (N=21) rated their ability to record daily glucose log as a “very useful” feature in patient portals [40]. Despite its usefulness, our study participants reported challenges in uploading BG results. While no specific recommendations were provided by study participants to address the challenge of uploading BG results, existing technology could be leveraged to address this problem. We submit that the design and adoption of portal features be expanded to accommodate remote communication and ensure interoperability with existing BG devices to allow for seamless uploading of BG data as well as summary data from widely used apps and wearable devices, such as for physical activity and diet. Linking these devices with patient portals can enhance transparency between clinicians and patients in relation to patients’ diabetes management [77]. Although wearable devices are now ubiquitous, linking them with patient portals may present a number of challenges that require creative solutions [78]. A typical challenge is the management of the huge data streams recorded by these devices that may require a substantial bandwidth to transmit and an expanded hospital IT infrastructure to host [79]. AI algorithms have been proposed as a way to extract and generate brief but clinically meaningful summaries from wearables that can be shared with clinicians and linked with portals. Sharing aggregate, preprocessed data as opposed to raw data can reduce clinician burden. In 1 study, informative BG reports from CGM devices were able to be remotely downloaded by clinicians and automatically added to patients’ EHR with just a few clicks [77]. While this integration is not currently widespread, it offers promise to address some of the concerns clinicians and patients have raised regarding portal use in adults with T2D. A summary of patient, clinician, and design-level implementation strategies is presented in Textbox 3.

**Summary of patient-, clinic-, and design-level implementation strategies for community health centers.**

**Patient level**

- Flexible portal training
  Combine in-person and remote sessions, including the use of prerecorded videos
  Screen for health literacy using single-item tools and determine the feasibility of using a portal versus other form of communication.
  Use infographics and simple educational materials for patient portal and type 2 diabetes education.
  Use the teach-back method for patients to demonstrate the use of patient portal.
  Encourage the involvement of family caregivers as proxy users, especially for patients with limited technology literacy.
  Provide easily accessible technical support for patient portal access issues

**Clinic level**

- Train clinic team to recognize the benefits of patient portal use and encourage them to raise or maintain awareness of patient portal among patients
- Implement a triaging system for patient messages
  Have a designated support team who is guided by an algorithm to triage new messages
  Consider incorporating artificial intelligence technology to identify high-priority messages and automatically route messages to the right clinician
- Monitor patient portal use and determine policies for nonuse and nonresponse to messages.
- Implement equitable portal reimbursement policies as relevant.
  Monitor and evaluate patients’ experiences and outcomes following the implementation of any portal billing policies
  Waive copays for portal-related billing

**Portal design level**

- Customize portal notifications to meet the needs of clinicians and patients
  Dashboard notification for clinicians
  Email or SMS text message notification for patients per preference
- Include language preference options
- Ensure seamless data interoperability with technologies, including continuous glucose monitoring
  - Presentation of clinically meaningful summary of blood glucose data and reports from wearable technologies

### Limitations

This study has several limitations. First, our sample size was relatively small, and our sample of adults with T2D had very limited use of the patient portal. For participants without portal experience, we explained the purpose of patient portals to access health information, schedule appointments, obtain medication refills, and communicate with their health care team. In subsequent research, exploring patient portal use among active users with T2D is recommended. Second, our sample was drawn from only 2 clinics, both of which are in Connecticut. Thus, our sample may not be representative of clinicians and adults with T2D who access care in CHCs across the United States. However, our goal was not to achieve representativeness but rather to provide preliminary evidence on the specific challenges to patient portal use in CHCs and how these challenges can be addressed.

### Conclusions

Adults with T2D and clinicians at CHCs continue to report pervasive challenges to patient portal use in CHCs. A lack of training and technical support for patients with low literacy to register and use the patient portal coupled with a lack of standardized implementation strategies to address the unique needs of patients receiving care at CHCs continue to impede efforts in achieving health equity in patient portal use. Implementation strategies recommended to improve health equity include portal training for those with low literacy, easily accessible technical support, a nurse triaging system for monitoring and responding to initial messages, algorithms for nurse triaging of chronic conditions, clinic policies for nonresponse to messages and reimbursement, customized notifications for patients and clinicians on new messages, and easy uploading of wearable technology data to the portal.

## Abbreviations

AI: artificial intelligence
BG: blood glucose
CGM: continuous glucose monitoring
CHC: community health center
CHW: community health worker
EHR: electronic health record
RA: research assistant
SDOH: social determinants of health
T2D: type 2 diabetes
